# Categorizing Foods by Relative Healthfulness: A Scoping Review of Front of Pack Labelling

**DOI:** 10.3390/ijerph182211980

**Published:** 2021-11-15

**Authors:** Shivneta Singh, Ashika Naicker, Sinenhlanhla Ntokozo Memela

**Affiliations:** Department of Food and Nutrition, Faculty of Applied Sciences, Durban University of Technology, 70 Steve Biko Road, Musgrave, Berea 4001, South Africa; ashikan@dut.ac.za (A.N.); SinenhlanhlaM@dut.ac.za (S.N.M.)

**Keywords:** canteen, front of pack (FOP) labelling, healthfulness, worksite, countries, intervention

## Abstract

Worksites are a suitable platform for employees to engage in behavioral change towards a healthy lifestyle by the modification of the food environment. Grading canteen foods at worksites into categories of relative healthfulness is an important indicator in the planning of food environmental interventions. However, in the absence of mandatory front of pack (FOP) labelling in South Africa, categorizing packaged and cooked food at worksite canteens is challenging. A scoping review was conducted on FOP labelling schemes to inform the selection of a FOP labelling scheme best suited for canteen foods at worksites in South Africa. The results of the scoping study, tabulated into a narrative summary, showed that there are several well-developed approaches to classifying foods by relative healthfulness through nutrient profiling and different forms of expression. It is recommended that because worksite canteen food sales in South Africa include both packaged and cooked food, and that a general test of various labelling schemes should be conducted to determine if a directional change is made towards purchasing healthier foods. Grading foods using interpretational aides such as an adapted FOP nutrition label to the South African context into categories of relative healthfulness can be a practical tool to inform food environmental interventions at worksite canteens and beyond.

## 1. Introduction

Non-communicable diseases (NCDs) have emerged as a worldwide pandemic in recent years, with incidences alarmingly greater in third world countries [1]. Non-communicable diseases account for 41 million fatalities annually, or 71 percent of all deaths [2]. Unhealthy diets are a major cause of mortality and disability across the world, with approximately 1 in every 5 fatalities, equating to 11 million deaths each year. Obesity and diet-related NCDs such as hypertension, cardiovascular disease, type 2 diabetes, and certain cancers are fuelled by the unparalleled availability, accessibility, and affordability of processed and pre-packaged foods [3].

Over the years, there have been significant changes in food systems, and diets have increasingly become more westernized in low to middle income countries (LMIC) [4]. As a consequence of the progress of multi-national food companies, the liberalization of international trade and investment in food and the rise in advertising of unhealthy food items, conventional raw-based diets are replaced by ready-to-eat, energy-dense, and non-nutritious meals or snacks [5]. Many of the ready to eat meals are highly processed which increases the risk of developing chronic diseases [6].

In South Africa, after the post-apartheid government allowed international trade and foreign direct investment, there has been a drastic shift in diets. Big Food companies (large commercial corporations leading the food and beverage environment) dominate the food supply with more affordable and inexpensive products [7]. This has led to a shift in the normative food culture, making more items accessible, which has catalyzed a shift in eating habits in rural and urban areas [8].

Society relies heavily on the working population [9]. Company sustainability and growth are directly influenced by the health of workers and are connected to national economic development, progress and social stability. The functioning and efficiency of individuals (usually 18–65 years) can be affected by many unhealthy behaviors. The World Health Organization (WHO) recommends that the workplace plays a vital role in health promotion [10]. There are many reasons why the worksite is an excellent location for health promotion; employees spend most of their waking hours at work, the working population is moderately healthy, the worksite is appropriate for long-term health interventions and follow-ups, and the workplace can provide participants with infrastructure and management support for lifestyle interventions. The United States Centers for Disease Control and Prevention (CDC) also supports health promotion services in the workplace through successful health promotion programs because of the benefits to employers, workers, employee families, and communities [11].

During the workday, many employees eat at least one or more meals, and therefore workplace interventions have the ability to affect a wide number of individuals, including people who are unlikely to engage in preventive health behavior [12]. Worksite interventions, in addition to boosting food intake at work, can positively impact employees and their families outside the work environment by secondary improvements in lifestyles [13]. Long term, they can also influence social norms around food choices and physical activity. In a recent review, it was noted that interventions targeting food quality or quantity, interventions targeting a client’s information, education, or motivation, and interventions targeting food choice at point of purchase have the potential to produce positive health related behaviors at worksites [14]. In this review, nine studies using FOP labelling significantly increased the sales of healthy food and beverages through traffic light labelling and healthy food logos [14].

Worksites have a wide range of choices to improve the supply and accessibility of nutritious food. FOP labelling assist consumers to make informed healthier food choices. Consumers require a simple and straightforward method for making healthy choices from the wide selection of items offered [15]. Concise FOP labels that are easily visible and take minimal time to examine are preferred by consumers [16]. While there are currently a range of FOP labelling schemes adopted internationally, simple, negative warning labels that easily determine unhealthy items tend to be the most successful for reducing the selection of energy-dense and highly processed food preferences thus far [15]. Warning labels are permitted to be displayed on the front of an item if the food and beverage does not match a particular nutritional standard [17]. These labels indicate whether the item is high in sugar, saturated or trans-fat, sodium, or calories, as required, which assist consumers in easily identifying unhealthy foods. FOP warning labels might similarly motivate food manufacturers to enhance the nutritional quality of their products. Nutrient-specific warning labels serve as summary indicators which present data on overall quality of a specific product [15]. This type of label also contains an interpretive system which depicts both numeric information and color-coded data which allows consumers to make a nutrient evaluative judgement when choosing foods. Table 1 indicates FOP labelling scheme adopted in various countries to classify food into categories of relative healthfulness.

Whilst the history of FOP labelling schemes is short, there has been a high level of activity globally. In 1989, the first interpretive FOP scheme was introduced [18]. The Swedish National Food Administration devised a symbol known as the “Green Key-hole” to help consumers select fat-free and fibre-rich food substitutes, exclusive of the need to read comprehensive nutritional labels. The Green Keyhole is displayed prominently on the packaging, but it can also be used on unpackaged items such as fish, vegetables, and fruit (such as berries) [18,19]. In 2004, the WHO Global Strategy on Diet, Physical Activity, and Health sparked the debate on interpretative FOP labelling as a policy tool to fight obesity [20]. In 2009, Nordic countries received permission to use the Green Keyhole symbol and cooperatively established the catchphrase “Healthy choices made easy” [21]. In 2013, the United Kingdom introduced a voluntary traffic light system, and since then it has become a voluntary mandate for producers under the Food Information Regulation [22]. In this system, a mix of color coding (traffic lights) and nutrition facts is used to indicate whether an item is high (red), medium (amber), or low (green) in fat, saturated fat, salt, and sugars, as well as how much energy (calorie and kilojoules) it supplies, at a glimpse. The Australian Health Star Rating (HSR), which came into effect in June 2014, is a nutrient-based FOP labelling scheme that rates products on a score of 0.5 to 5 stars for their proportion of ‘risk’ and ‘positive’ nutrients [23]. It allows consumers to quickly compare similar pack-aged meals and select the healthier alternative. The number of stars is determined by the amount of caloric, saturated fat, salt, and sugar in packaged foods as well as the amount of fibre, protein, fruit, vegetable, nut, and legume content. Health Star Ratings also incentivize food producers to modify their products to increase their star rating, which may result in consumers having access to healthier packaged meals [24]. Chile’s Food Labelling and Advertising Law was introduced in 2016, the first nationwide regulation to necessitate FOP warning labels, limit marketing aimed at children, and prohibit sales of all products that consist of added sugars, sodium, or saturated fats that surpasses nutrient or calorie cutoffs in schools [25]. The Nutri-Score label was first established as a voluntary label in France in 2017 and has subsequently been adopted by a few other countries in Europe [15]. The Nutri-Score is a summary indicator of product healthfulness which incorporates a color spectrum similar to traffic light labels, as well as letter grades. The summary score is based on a nutrient profile model that considers the health hazards and benefits of product constituents (for example, fibre, legume, nut, protein, fruit, and vegetable). In South Africa, between 2013 and 2017, the draft Regulations pertaining to the labelling and advertising of foodstuffs (R.429) were established for voluntary FOP labelling which advocated traffic-light nutrient labelling, with red, yellow, and green indicating the quantities of vital nutrients such as energy, total sugar, fat, saturated fat, and total sodium [26] but never endorsed. Currently in South Africa, Guideline Daily Amounts (GDAs) is a voluntary labelling system designed to assist consumers in making smart food choices [27]. It provides a summary of a product’s percentage of energy and important nutrients per serving. It specifies the amount of energy, fat, saturated fat, sugar, and sodium in the product. Guideline Daily Amounts are computed based on an average diet and can assist consumers in understanding what is in the food they consume. Guideline Daily Amounts recommendations are not determined for an individual’s nutrient needs, but rather as a baseline for the general population, because consumers differ in various aspects, for example, height, age, and gender. Consumers, especially those with poor education, have a difficult time deciphering nutritional information from the GDA labelling scheme, and would prefer a simpler method of presenting this information that would aid them in making a quick assessment of a product’s nutritional characteristics [28]. Presently, policies are being reviewed in South Africa to determine what FOP labelling scheme will be most suitable for South African consumers. As more countries work towards choosing and implementing the most appropriate FOP labelling scheme, more recently in 2020, the WHO published a manual to guide countries in implementing an effective FOP nutrition labelling scheme using a five-step process for developing and implementing an evidence-based FOP labelling scheme to assist consumers in making better food choices [29].

The South African Pioneer Worksite Intervention Study, made up of three phases, aims to measure the effectiveness of a canteen and behavioral intervention on cardio-metabolic risk at a worksite in South Africa. This scoping review forms part of phase 2 of the South African Pioneer Worksite Intervention Study. Phase 1 involves the recruitment of a suitable worksite, phase 2 involves the development of an acceptable, appropriate, and feasible worksite intervention that will inform the design of the food environment and behavioral intervention, and phase 3 involves the implementation of the worksite intervention to lower cardio-metabolic risk in South Africa. In the absence of mandatory FOP labelling in South Africa, the aim of the scoping review was to guide the selection of a FOP labelling scheme best suited for categorizing packaged and cooked canteen foods sold at worksites in South Africa. 

## 2. Materials and Methods

A scoping review was carried out to gather data from empirical findings on the categorization of healthy foods through FOP labelling schemes. The reporting of this analysis was influenced by the Preferred Reporting Items for Systematic Reviews and Meta-Analyses (PRISMA) extension for Scoping Reviews (PRISMA-ScR) guidelines. The search strategy included keywords such as “endorsement logo”, “categorizing food”, “front-of-package labelling”, “nutrient profiling”, “traffic light labelling”, “Nutri-score”, “food choice”, “canteen”, “worksite”, “countries”, and “food symbols”. PubMed, Scopus, and Google Scholar were the three databases used to search for relevant articles. All papers, including titles and abstracts, were imported into an endnote database and duplicates were removed. One independent reviewer screened the article’s titles and abstracts using a pre-specified inclusion criterion. The inclusion criteria included population group (adults and employees, both males and females), type of environment (food environment, worksites, and canteens), language (English), and effectiveness of the grading system. Further information was extracted based on studies that have previously used FOP symbols/logos to categorize food into healthfulness. The studies included were not confined to the worksite and canteen but also included studies reporting on the effectiveness of FOP labelling. Studies based on FOP labelling targeting children (below the age of 18 years), unpublished articles, studies that include FOP labelling that has not been implemented or adopted by the country, and non-English articles were excluded from the review.

## 3. Results

**Figure 1:**
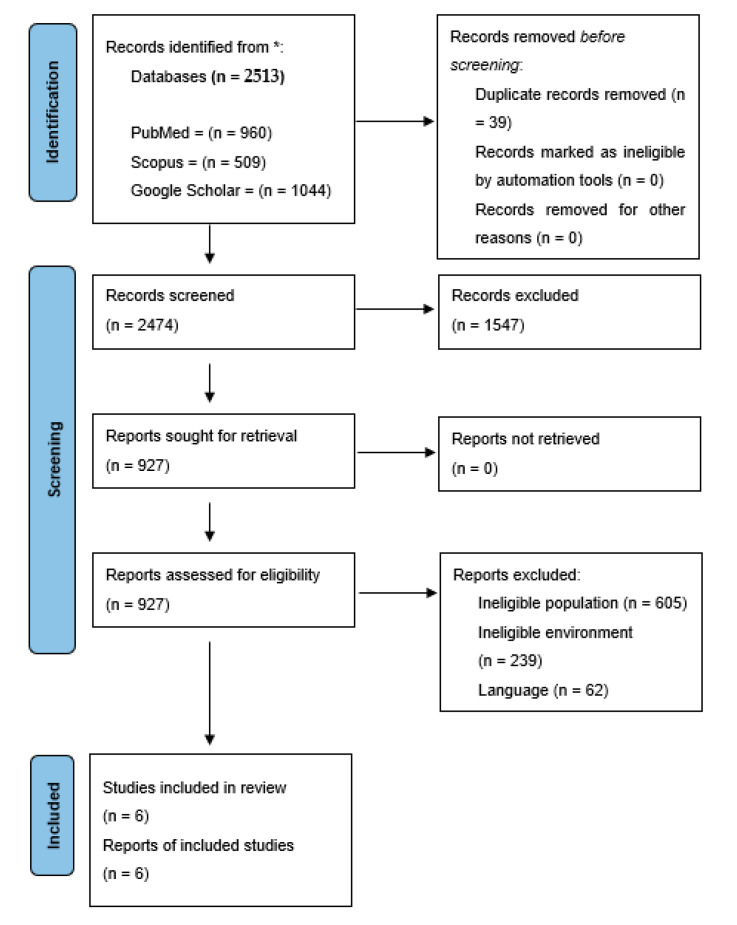
Preferred Reporting Items for Systematic Reviews and Meta-Analyses (PRISMA) flow [30]. * Consider, if feasible to do so, reporting the number of records identified from each database or register searched (rather than the total number across all databases/registers).

Several articles (*n* = 2513) were identified and screened after excluding duplicates (*n* = 2474). Overall, 1347 articles were excluded from the study because their abstracts and titles did not match the qualifying criteria (Figure 1). The grounds for withdrawing the other studies (*n* = 921) were that they did not meet the requirements for inclusion. A total of six articles was used in the qualitative analysis after a full-text review of the remaining articles. The six studies in the final review included countries such as Europe, France, Australia, Chile, Denmark, and the Eastern Mediterranean Region. Table 2 summarizes the studies reviewed to categorize foods according to the FOP labelling scheme.

## 4. Discussion

Food labeling is defined as any written, printed, or graphic material that is featured on the label, accompanies the food, or is exhibited near the food, including that with the aim of encouraging its sale or discarding [36]. Food labels have two purposes: to inform consumers and to help in the sale of the product [37]. However, food labels have developed over the years in terms of the information they represent due to the influence of food legislation, food corporations, merchants, public authorities, and consumers. The component of a food label that expressly discloses nutritional content is known as nutrition labelling [38]. Nutrition labelling is functional, according to the Codex Alimentarius, when it offers information about a product to assist consumers in making healthy eating decisions [39].

There is a substantial and expanding body of research on nutrition labels, involving multiple literature studies that have been undertaken since 1991 [40]. The results were largely comparable, with self-reported utilization of nutrition labels being common. Consumers, on the other hand, have had difficulty interpreting quantitative information on labels, and some have considered varied nutrition label forms, and thus too much information supplied on the label, to be perplexing. Consumers preferred visual content, such as a logo, over the typical nutrition information table [41]. According to a comprehensive study conducted by Campos et al., nutrition labels were considered to be a very reliable source of nutrition information as many consumers utilize nutrition labels as a guide when purchasing food items [40]. The usage of nutrition labels, on the other hand, differs greatly amongst populations as results indicated that younger adults and middle-aged females are more likely to utilize nutrition labels. The findings from an investigation conducted by Bosman et al., highlights that consumers in South Africa are able to deduce and interpret nutrition information on food labels to a certain level [42]. In contrast, other consumers expressed doubts about their comprehension of the information presented. Consumers also had difficulty deciphering nutrition labels because of the font size of the nutrition information and the wording included in the ingredient list [43]. Consumers do not read labels for a variety of reasons, including disinterest, time constraints, cost, and inveterate buying. The taste of a product is more pertinent to some consumers than its nutritional value [44]. This underscores the importance of educating consumers on how to make healthier food choices while utilizing the information supplied on food labels, but within the constraints of the identified components, such as educating consumers on how to compare nutrition information presented on food labels for products within a particular price category.

In the six studies reviewed in this article, four studies were based on endorsement logos [19,32,33,35]. Endorsement logos are one of the four main types of FOP labelling [19]. This provides information on the nutrient levels combined to provide an overall assessment of healthfulness and positive evaluative judgment on better-for-you foods. A product may only be eligible to display an endorsement symbol if it meets a nutrition standard and nutrient cut-off points binary. Front-of-packaging labelling can be useful tool to communicate simple nutrition information to consumers, increasing their ability to make healthy food choices and therefore creates a rise in point-of-sale. According to the study conducted by Mork et al., two out of three retail stores had a positive sales outcome regarding the purchase of Green keyhole-labelled products (20% increase) [33]. Interpretative summary indicator labels such as graded summary system (Nutri-score and health logos) are useful to consumers who want to compare various substitutes when purchasing to select the healthiest product. Research shows that interpretative labels are more effective than reductive labels when motivating consumers to make healthier food choices [45]. Analysis by Grunert and Wills indicates that consumers typically prefer the convenience of overview labels, but endorse formats that also provide them with sufficient detail on the product’s nutritional content [46]. According to the findings of a study undertaken in the Western Cape [47], focus group discussions’ (FGDs) participants were optimistic regarding a single Health Endorsement Logo (HEL), suggesting that it would render food labelling less complicated since the various HELs used were not understood. Participants suggested that terminology related to ‘better choice’ or ‘healthy choice’ and health and/or food related photos or symbols should be used in the logo. HEL was planned and tested by consumers. After further testing, three prototypes were sent to the national health department to be considered for implementation as a method to assist in resolving the high incidence of non-communicable diseases in South Africa. The limitations for endorsement logos include population groups and geographical aspects due to ethnic groups, literacy levels, language, and access to resources. Studies should include the evaluation and effectiveness of endorsement logos for acceptability and comprehension in various regions to accommodate all types of consumers.

For reasons related to disease prevention and health promotion, nutrient profiling is defined as the science of classifying or ranking foods by their nutritional composition [48]. Nutrient profiling can be used for a wide variety of purposes, including children’s food promotions, health and nutrition statements, logos or icons for product labelling, knowledge and education, the supply of food to public institutions, and the use of economic instruments to direct food consumption. Nutrient profiling can, for example, be used to create requirements for food descriptions falling into two key types: descriptions that apply to food nutrient levels (for instance, high in fat, sugar, or salt) or descriptions which directly refer to the effects of eating food on the health of a person (example, “good for you”). According to Table 2, two out of the six studies were based on nutrient profiling [31,34]. The first study resulted in the development of nutritional profiles by the United Kingdom British Food Standards Agency Nutrient Profile System (FSA-NPS), which used a simple scoring system. The scoring system was implemented by assigning a point to 100 g of nutrients present in the food item [31]. The second study identified the effectiveness of the HSR on consumers’ decision making process [34]. The results indicate that consumer’s nutrition knowledge plays a vital role on their buying decisions. Products exhibiting a mandated labelling such as the HSR enhance consumers’ capacity to identify and choose healthier food items even if they did not view the BOP nutrition information. In addition, a recent study conducted by Kupirovic et al. showed that all front-of-package nutrition labelling systems will make it easier for customers to make healthier decisions so they can follow dietary recommendations and distinguish within a category between healthy and less healthy items [49]. The limitations for nutrient profiling include not enough evidence as to which labelling scheme is most suitable and successful. The most suitable front-of-packaging labelling varies from country to country due to national backgrounds [32], therefore policy makers need to decide and select the scheme that is most appropriate to their distinctive nationality.

## 5. Limitations and Future Directions of the Study

There is limited evidence on the most suitable FOP labelling scheme. Comparative studies should be conducted to assess multiple core aspects of labelling systems instead of being simple variations of a certain geographical format. For instance, if a warning system is being proposed, it should be compared to a graded label (for example, nutrient-based—Multiple Traffic Light, or summary—Health Star Rating System or the Nutri-Score), and conversely.

## 6. Conclusions

Front-of-pack nutrition labelling is one of the evolving structural initiatives undertaken to enhance the food environment to address the steadily rising burden of diet-related NCDs. It is an inexpensive method that delivers simple and at-a-glance nutritional information to support consumers in making informed food choices at the point of purchase. In attempt to address the NCD crisis in South Africa, an intervention, such as the promotion of a healthy diet through the supply of proper nutrition information on food labels, along with consumer education to help understand nutrition labels, is required.

Grading foods into categories of healthfulness through evidence of key nutritional dimensions is a practical tool to inform food environmental interventions that may assist in public health promotion by influencing consumer choice in workplace canteens and beyond. It is recommended that because worksite canteen food sales in South Africa include both packaged and cooked food, a general test of various labelling schemes should be conducted to determine if a directional change is made towards purchasing healthier foods. Grading foods using interpretational aides adapted to the South African context into categories of relative healthfulness can be a practical tool to inform food environmental interventions at worksite canteens and beyond.

## Figures and Tables

**Table 1:** Front of Package labelling scheme used in various countries to classify food into categories of relative healthfulness.

Form of Expression	FOP Labelling Scheme	Logo/Symbol with Graphic Example	Brief Description	Nutrients/Ingredients Included	Country Where the System Is Used or Proposed
Summary Labels					
Simple	Endorsement Logo	Choices Logo 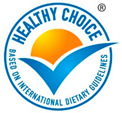	Green logo with a “healthy choice” text that displays healthy options (including bread, milk, fruit, and vegetables) within specific categories essential to a healthy diet. Blue logo with “conscious choice” text to help customers make better decisions for non-basic items.	Saturated and trans-fat, sodium, added sugar across all foods. Energy criteria for non-basic food groups.	Poland, Belgium, Czechia, Netherlands (until 2016)
	Endorsement Logo	Green Endorsement Logo 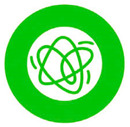	Squiggle within a green circle.	Sugar, sodium, saturated fat, and fiber (criteria not yet available).	Israel
	Endorsement Logo	Healthy Living Guarantee Mark 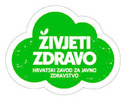	Green cloud with text “Live Well”.	Total fat, saturated fat, sugar, sodium, and fiber.	Croatia
	Endorsement Logo	Heart Symbol 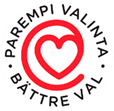	Heart symbol with encircling text “Better choice”.	Total fat, saturated and unsaturated fat, sodium, sugar, and fiber.	Finland
	Endorsement Logo	Keyhole Logo 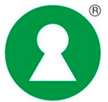	Green with a white keyhole, followed by the registered trademark symbol.	Total fat, saturated and trans-fat, added sugar, salt, dietary fiber, and whole grains.	Iceland, Lithuania, Denmark, Norway, Sweden
	Endorsement Logo	Protective Food Logo (Little heart Logo) 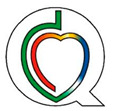	List below heart symbol gives the product’s specific nutritional properties that make it a healthier option compared with other food products in the same category.	Fat, ratio of fatty acids, salt, added sugar, energy, and fiber.	Slovenia
Nutrient-Specific Labels					
Color-Coded	Nutrient-specific interpretive system	Color-coded %RI (Recommended intake) 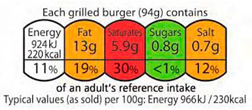	Traffic light color coding: Interpretive (color) and non-interpretive (%RI). Color coding indicates high (red), medium (amber), and low (green) levels of negative nutrients. Energy is depicted in greyscale.	Energy, total fat, saturated fat, sugars, and salt.	United Kingdom, Portugal, Ireland
Warning Labels	Nutrient-specific warning label	Red warning label 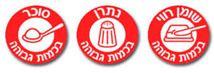	Single symbols used for sugar (spoon), sodium (saltshaker) and saturated fat (solid fat and knife), with text “high in nutrient”. Back of pack displays the amount of calories, sugar, sodium, and saturated fat in bold font.	Sugar, sodium, and saturated fat.	Israel
	Nutrient-specific warning label	The Chilean System 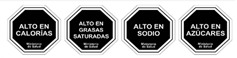	The words “alto en…” (“high in…”) calories, sugars, saturated fats, or sodium are written in a black octagon with white borders on the front of the food or beverage package.	Saturated fats, sodium, calories, and sugar.	Chile, Uruguay, Peru, Israel, Brazil, Canada, India.

Adapted from Jewell and Kelly (2019) [19].

**Table 2:** Review on FOP labelling scheme studies to categorize food by relative healthfulness.

FOP Labelling Scheme	Author, Year, Location	Population	Results of Study	Limitations
Endorsement Logo	Jewell and Kelly (2019) [19] Europe	13 countries: Croatia, Czechia, Denmark, Finland, France, Iceland, Israel, Lithuania, Norway, Poland, Slovenia, Sweden and the United Kingdom, Belgium, and Netherlands. Study population not reported.	The aim of the logo is to suggest options that are better for you but provide no clear details to suggest whether a product is unhealthy. Front of packaging labelling (FOLP) policies were supported by government in three countries that provided directive details on product healthiness. This included nutrient-specific warning labels, a summary indicator system providing information on the overall nutrition quality of a product, and an interpretive system providing both numerical and color-coding information on the contribution a food makes to a nutrient ‘s prescribed daily intake.	Lack of information on formal provisions for the assessment of FOPL policies as part of label implementation, while scholarly reviews have provided proof in encouraging the consumer and reformulation objectives.
Summary indicator system: Nutri-Score information system	Hurtado et al. (2020) [31] France	Study population not reported.	The nutritional profiles developed by the United Kingdom British Food Standards Agency Nutrient Profile System (FSA-NPS) were used as the for the scoring criteria for the nutritional profiles of Food Standards the Australian and New Zealand, the model of nutritional profiles in South Africa and the model of nutritional profiles in Ireland. The FSA algorithm consists of a basic scoring system in which points are assigned on the basis of per 100 g of product nutrient material.	FOPL development: it may not be considered acceptable to use a dichotomous scoring system (with binary scoring suggesting the definition of good and bad food products). Taking this into account, Santé Publique (Public Health) France, in collaboration with the University of Paris, created five nutritional quality categories based on the British FSA-NPS, in order to ensure a high degree of discrimination within each food and beverage division, while retaining a core category in order to avoid classifying food items as good or bad.
Nutrient-specific interpretive system: Traffic Light Labelling	Al-Jawaldeh et al. (2020) [32] Eastern Mediterranean Region	Study population not reported	Three countries in the area have been reported as introducing front-of-pack nutrition labelling schemes, and three more schemes are under progress. In the area, the regimes listed fell into three categories: The nutrient-specific traffic light labelling (Islamic Republic of Iran, Kingdom of Saudi Arabia, United Arab Emirates); The Nutri-Score summary graded label (Morocco); Health or endorsement logos (Abu Dhabi and Tunisia).	In real world environments, there is not enough testing. No conclusive proof exists as to which particular scheme is most successful. The most suitable FOP labelling scheme can differ from country to country, so policy makers need to select the scheme that is most appropriate for their unique national background.
Endorsement Logo—Keyhole logo	Mork et al. (2017) [33] Denmark	Males older than 35 years old with poor educational standards	In two out of three supermarket outlets investigated, the initiative had a positive impact on sales of keyhole labelled items. Sales of keyhole branded goods increased by approximately 20% in these two retail chains. There was a slight decline in sales of keyhole branded goods in the third chain. The impact varied considerably between categories of goods. Interview data analysis found that shoppers with poor education had a greater likelihood of mentioning health as a purchase motive by the end of the campaign, and there was a higher general propensity to search for nutrition information.	The findings are based on a few selected stores examined. As the frozen ready to eat meal counter was not very well visited, the observation/interview portion of the analysis is restricted by the range of product categories and may be one of the categories where a nutrition label may have a reverse effect. The experiments were also carried out within a short time frame, and it was therefore not possible to calculate the long-term impact of the initiative.
Nutrient-specific interpretive system: Australian Health Star Rating System	O’Connor and Anderson (2019) [34] Australia	Males and females aged 17–83 years old from the community and psychology students from the Queensland University of Technology.	The purpose of this study was to see how the Australian HSR effects consumer decision-making in various comparative processing scenarios. Individuals were asked to complete six binary forced-choice comparisons wherein the appearance of the HSR and the nutritional status (high or low) of the cereal products were both changed. Participants were also asked to rate their willingness to buy the products. As opposed to prior research, consumers did not interpret the existence of the HSR as a sign of a healthier alternative. This indicates that the level of cognitive processes required to assess the HSR system is suitable for successful decision-making. When evaluating a product excluding a HSR label to a product including a HSR label, individuals who did not review the back-of-pack (BOP) nutritional information were more likely to make less informed choices. Irrespective of BOP viewing, consumers’ capacity to choose healthier items was enhanced when both products exhibited a HSR (namely a mandated labeling). This shows that consumer decision-making is influenced by the sort of comparison environment. Consumers’ propensity to purchase products with low and high nutritional content was also found to be affected differently by nutrition knowledge.	The participants in this study were predominantly women, aged 17–24, with secondary school education. One of the limitations in the study is that the results may not be reflective of the average working-class Australian household. Future studies should include a more diverse group as results that are used to inform public policies must be generalizable. Another limitation is that when respondents had no apparent product preference, the forced-choice method may have inadvertently altered consumer decision.
Endorsement Logo	Reyez et al. (2019) [35] Chile	Study population not reported.	Information on general text, for example, short wording and design characteristics (such as use of the logo, use of red or black colors), was presented in the literature review and qualitative stage; 15 prototypes were produced and quantitatively evaluated on the basis of the selected characteristics. A black and white stop sign and a black-and white hand were preselected in the first survey. In the second survey, in terms of visualization, intention to buy and willingness to change the planned purchase, the stop sign saying ‘Excess of <nutrient>‘ had a considerably higher score than the hand.	The FOP warning label suggested in this article was introduced by the Chilean Minister of Health by replacing the words’ Excess of ‘with’ High in ‘because the language of the legislation did not permit the use of’ Excess.’ A communication campaign to present the latest alert message was launched to ensure that individuals correctly interpreted the significance of ‘High in’. The use of the term ‘High in’ as a beneficial food attribute (that is, high in vitamins) was forbidden.

## Data Availability

Not applicable.
